# P-18. Feasibility of PO Step-Down Therapy in Streptococcal Bloodstream Infections: Insights from a Cancer Institute

**DOI:** 10.1093/ofid/ofaf695.249

**Published:** 2026-01-11

**Authors:** Erin Bybee, John Lloyd, Leslie Lin, Brittni Clopton, Abdallah Almawazreh, Lea M Monday

**Affiliations:** Wayne State University School of Medicine, Warren, Michigan; Wayne State University School of Medicine, Warren, Michigan; Detroit Medical Center, Detroit, Michigan; Detroit Medical Center, Detroit, Michigan; Detroit Medical Center, Detroit, Michigan; Wayne state University School of Medicine, Detroit, Michigan

## Abstract

**Background:**

Data suggests intravenous (IV) to oral (PO) antibiotic de-escalation is appropriate for uncomplicated streptococcal bloodstream infections (BSI). However, significant heterogeneity exist in exclusion criteria among studies (table 1). Data on PO treatment for complicated BSI is sparse, and none exist exclusively in oncology patients. We evaluated rates and outcomes of PO step-down therapy in patients with streptococcal BSI at our NCI-designated cancer institute to assess the feasibility of a quality improvement (QI) project aimed at optimizing PO therapy in BSI.

**Figure:**
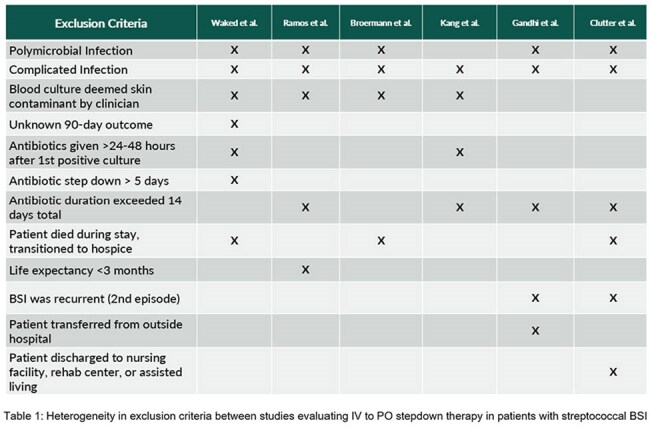

**Figure:**
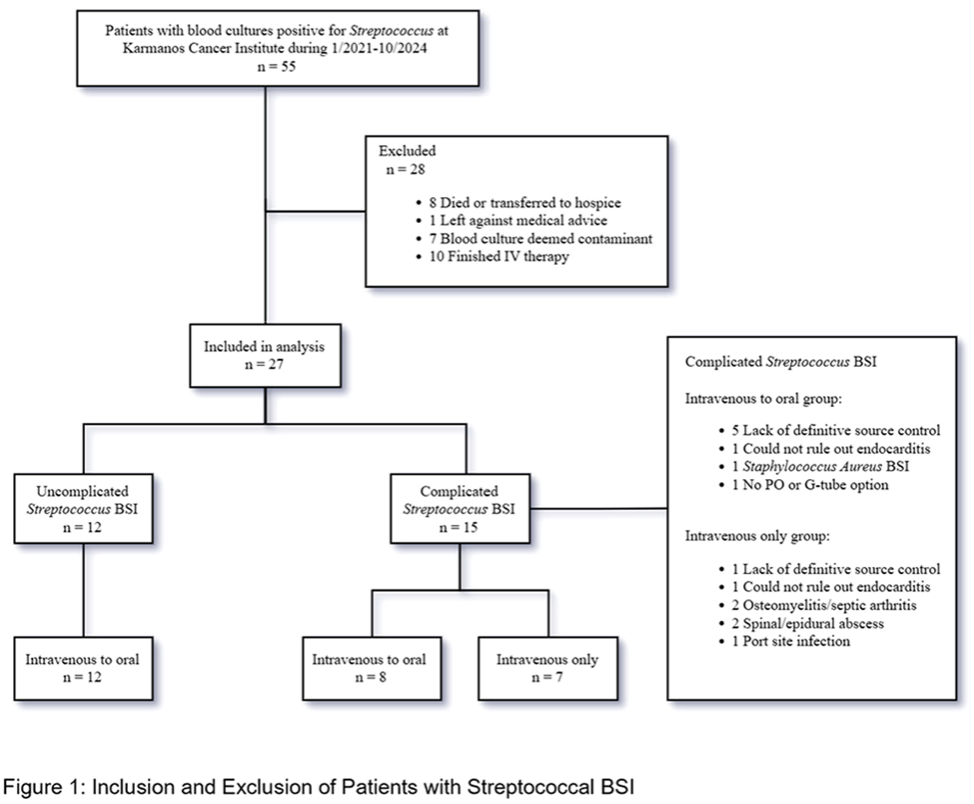

**Methods:**

This was a retrospective study of adult patients with streptococcal BSI (Jan.2021-Oct.2024) at Karmanos Cancer Institute; Detroit, MI. Patients were excluded for: inability to take PO medication, concomitant *S. aureus* BSI, completion of IV course inpatient, death/hospice, or suspected contaminant. Patients with polymicrobial BSI (except *S. aureus*), suspected endocarditis, osteomyelitis, septic arthritis, persistent bacteremia, unclear infection source, or lack of definitive source control, were considered complicated BSI and included. All other patients were considered uncomplicated BSI. Primary outcome was the rate of PO step down therapy in uncomplicated and complicated BSI. Secondary outcomes included 30-day bacteremia recurrence, length of stay (LOS), and *C. difficile* infection (CDI).

**Figure:**
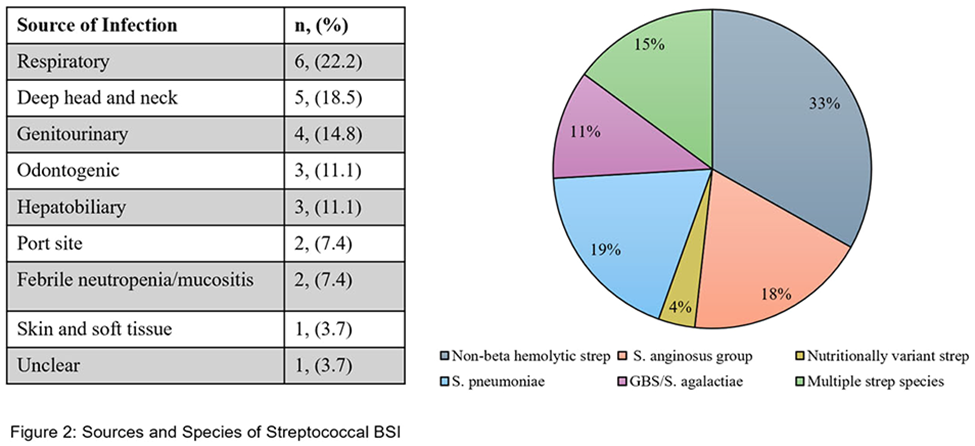

**Results:**

55 patients had streptococcus BSI; 28 were excluded, 27 met inclusion criteria (12 uncomplicated and 15 complicated) (Fig 1). BSI source was most commonly respiratory (Fig 2). All 12 patients with uncomplicated BSI were transitioned to PO therapy at discharge. 7 complicated BSI patients were discharged on IV antibiotics. 8 patients with complicated BSI were given PO therapy for discharge. Median LOS was shorter in patients discharged on PO (6 vs 10 days). One patient discharged on PO returned with CDI. No patients in either group had BSI recurrence.

**Conclusion:**

IV-to-PO step-down therapy for streptococcal BSIs was commonly practiced at our institution even in patients with complicated infections and did not lead to bacteremia recurrence. Given the lack of IV therapy use for uncomplicated BSI, further QI projects to investigate this may be low yield at cancer centers where practices are similar.

**Disclosures:**

All Authors: No reported disclosures

